# Recovery of Renal Function after One-Year of Dialysis Treatment: Case Report and Registry Data

**DOI:** 10.4061/2010/817836

**Published:** 2010-06-17

**Authors:** Ingela Fehrman-Ekholm, Ann-Charlotte Bergenhag, Olof Heimburger, Staffan Schön

**Affiliations:** ^1^Dialysis Unit, Sophiahemmet, Karolinska University Hospital, P.O. Box 5605, 114 86 Stockholm, Sweden; ^2^Department of Renal Medicine, Karolinska University Hospital, P.O. Box 5605, 141 86 Stockholm, Sweden; ^3^Swedish Renal Registry (SRR), Medicinexp, plan 5, Länssjukhuset Ryhov, 551 85 Jönköping, Sweden

## Abstract

*Objective*. Uncertainty has arisen as to whether renal function can be recovered from after long-term regular dialysis treatment. We therefore conducted an analysis and scrutinized one patient report. *Material and Methods*. Swedish registry of patients with kidney disease and one patient case. *Results*. 39 patients (0.2%) from the Swedish registry comprising 17590 patients who commenced RRT (renal replacement therapy) between 1991 and 2008 had recovered from renal function after more than 365 days of regular dialysis treatment. The most common diagnosis was renovascular disease with hypertension but a large group had uremia of unknown cause. HUS, cortical/tubular necrosis, and autoimmune diseases were also found. The mean treatment time before withdrawal was 2 years. *Conclusions*. A small number of patients recover after a long period of regular dialysis treatment. One could discuss whether it is difficult to identify patients who have recovered while undergoing regular dialysis treatment. Regular monitoring of renal function may be important.

## 1. Introduction

Recovery of renal function in end-stage renal disease in patients receiving renal replacement therapy has been described as occurring in 0.3%–8% [1–3]. A recent study from Australia revealed that recovery occurred in 1% of the dialysis population and there was no difference between PD or HD [4]. In the literature the cases of recovery have included various diagnoses: surgery after total renal artery arteriosclerosis, cholesterol crystal embolism, FSGS secondary to HIV, secondary oxalosis, and accelerated hypertension. 

Having experienced a patient who was taken off from dialysis treatment after 15 months and who 18 months after this process is still in no need of regular dialysis motivated us to present the case and scrutinize the Swedish registry to find similar cases with dialysis treatment of more than one year followed by withdrawal. We considered it important to establish the diagnoses behind the diseases that could abate after such a long treatment period.

## 2. Results

We present our case report.

### 2.1. Case Report

A healthy 49-year-old man developed acute problems with headache and vomiting. He was admitted to hospital in November 2006. It was found that his blood pressure was high measuring 228/138 mm Hg in the left arm and 205/145 in the right arm, kidney function poor with creatinine 997 *μ*mol/L, anemia with Hb 90 g/L, and thrombocytopenia with platelets 40 × 10^9^/L. Further investigation revealed that lactate dehydrogenase (LD) was elevated to 38.2 *μ*cat/L (NV < 3.5 *μ*cat/L) and aspartaminotranspheras (ASAT) to 1.67 *μ*cat/L (NV < 0.76 *μ*cat/L). The peripheral blood smear showed schistocytes and spherocytes and several reticulocytes. The clinical diagnosis was HUS and plasmapheresis was administered. The substitution fluid was plasma. However, the patient did not tolerate the treatment and became anuric with pulmonary edema. His creatinine level had now risen to 1247 *μ*mol/L. Hemodialysis was started acutely on the 12th of November and after that provided regularly three times per week. On the 23rd of November a kidney biopsy was performed. It showed severe vascular changes and several collapsed glomeruli compatible with the diagnosis of thrombotic microangiopathy and malignant hypertension. Immunofluorescence was negative.

The dialysis treatment continued and blood pressure treatment included 4 drugs (enalapril, candesartan, felodipine and metoprolol). The diuresis started to reappear and in January 2007 it was measured to 1700 mL between two dialysis schedules. He had received a central dialysis catheter on the 15th of November and an AV fistula was created in the end of January which however thrombotized. The size of the kidneys was not measured at this time point. In February 2007 an AV fistula was constructed in the upper arm, which worked perfectly and still is functioning well.

In May 2007 kidney transplantation was discussed and his sister was investigated as a donor. However, it turned out that she had had several DVTs and was thus deemed unsuitable for donation. The coagulation investigation of our patient indicated that he was heterozygote for APC resistance. The patient told us that he had good diuresis and therefore a measurement of kidney function was performed. A 48-hour iohexol clearance measurement showed a value of 9.7 mL/min/1.73 m^2^ body surface. The transplantation plans were changed. The dialysis schedule was instead reduced to twice per week. The patient's blood pressure was stable and well controlled by the four drugs and diuretics (Table 1).

In September 2007 it became possible to withdraw the EPO treatment due to a stable hemoglobin value of around 129 g/L.

In January 2008 a new 48-hour iohexolclearance measurement yielded a value of 13 mL/min/1.73 m^2^ body surface. The dialysis treatment was further reduced to once a week.

On the 13th of March, which was 514 days after the start of renal replacement, the dialysis treatment was withdrawn completely. The patient was monitored every week. He felt very well. In May 2008 the iohexol clearance was 16 mL/min/1.73 m^2^ body surface. The time between checkups was now extended to 2-3 weeks. S-creatinine varied between 320 and 410 *μ*mol/L.

In August the patient went to Thailand for a 4-week vacation. When he returned he was hypotonic and acidotic with uremic signs. His s-creatinine was 509 and urea was 33.4. After a couple of dialysis treatments and fluid he recovered from his symptoms. 

In February 2009 he had a prolonged infection with bronchitis. The urea had increased to 40.5 mmol/L and creatinine to 386 *μ*mol/L. CRP was 71 mg/L. He was given one dialysis treatment and antibiotics and recovered quickly.

In March 2009, thus one year after withdrawal of dialysis, his renal function was 21 mL/min/1.73 m^2^. His blood pressure (BP) was well controlled with BP 120/60. The s-creatinine was 288 *μ*mol/L and electrolytes were good. Cystatin C-estimated GFR was 22 mL/min/1.73 m^2^. In October 2009, thus 18 months after withdrawal, the renal function measured with iohexol clearance was 23 mL/min/1.73 m^2^, the Cystatin C-estimated GFR was 24 mL/min/1.73 m^2^, s-creatinine was 212 *μ*mol/L, and the patient was in very good health and working full time. His blood pressure was 123/73 and urine albumin/creatinine ratio 6.3 mg/mmol.

The only disturbing factor was the slightly elevated LD, which was 4.6 *μ*cat/L, and elevated ASAT 2.76 *μ*cat/L. The CRP was normal, and the hemoglobin values were around 128 g/L, with no signs of hemolysis. A hepatic specialist stated that the enzymes are probably from muscles and most definitely not from the liver.

## 3. Swedish Renal Registry (SRR) Data

 We found 39 patients who had recovered from their renal function, which was defined as dialysis for more than 365 days followed by recovery (SRR, http://www.snronline.se/). The data are presented in Table 2 and reveal that recovery occurred in 14 women and 25 men after 383–2081 days of dialysis treatment. Mean dialysis time was 726 (SD 360) days. A total of 29 patients were treated by means of HD, six with PD, and four with both PD and HD for various lengths of time. The largest groups were those with renovascular disease with hypertension (eight) and chronic renal failure of unknown causes (seven). Of the known diagnoses the most common were hemolytic uremic syndrome (four) and cortical/tubular necrosis (four). Other diagnoses included cholesterol embolism (two), polyarteritis nodosa (two), sclerodermia (two), crescent glomerulonephritis (two), and SLE (two). All patients were alive 3 months after withdrawal, thus it was not done to stop ESRD treatment before impending death.

Time without dialysis treatment after withdrawal ranged from 84 to 6431 days and the mean value was 1415 days, that is, 3.9 years.

## 4. Discussion

The Swedish registry revealed that 0.2% of patients recovered from renal function after more than one year of regular dialysis treatment. It may seem a low number. The Swedish Renal Registry (SRR) has been working since 1991. All units performing dialysis and/or kidney transplantation in Sweden report to the registry. Validation of the registry has shown a high accuracy and few patients are missed to be reported. Patients are supposed to be reported as soon as they enter renal replacement therapy by the local nephrologist (registry keyman). The basic criterion for a patient to be reported is that the renal insufficiency is regarded as chronic and based on a chronic kidney disease. When/if a patient has regained renal function, the keyman also reports this to the registry as soon as possible. The registry quality has been maintained by repeated feedback reports to the keymen, by yearly cross-sectional studies of the dialysis population, and by estimation of the number of unknown cases (validation). SRR uses the ERA-EDTA coding system. The patients' identities are known to us but the medical records have not been scrutinized. 

If the rate of recovery from ESRD in the Swedish registry is regarded as low, this could be due to distinct criteria for chronic disease when entering the registry. Data on patients regarded as having an acute renal failure are deleted from the registry.

The data demonstrated that renovascular disease with hypertension was the most common diagnosis and obviously the kidneys can recover with good blood pressure control during dialysis treatment. However, a majority of the patients had no clear diagnosis that explained the reason for the renal failure. The most striking finding was the long renal replacement treatment time, the longest being 5.7 years and the mean treatment time being 2.0 years before renal replacement therapy was withdrawn. Is it difficult to stop dialysis treatment, especially after a long time? Have the kidneys recovered without it being detected? The study by Agraharkar et al. also shows that GFR based on creatinine clearance data at withdrawal was high, 29 mL/min on average with a range from 9 to 51 mL/min [1]. 

In our case report the diagnosis was thrombotic microangiopathy (TMA) with severe hypertension. The patient was classified as belonging to the group of renovascular diseases with hypertension, which is the largest group. Once his blood pressure had stabilized, the urine production started and the recovery process seemed to progress. A recovery phase of over six months has been described in acute hypertensive diseases by Yaqoob et al. [5]. 

Our own experience was that it is difficult to detect changes in renal function in dialysis patients. When we started to measure renal function with the injection technique, we found good values, which at first we did not believe to be accurate. However, regular measurements and the use of different methods for assessing renal function made us convinced. Both Cystatin C for estimating renal function and iohexol clearance for measuring function were used together with creatinine clearance. There was also a fear of stopping dialysis due to the risk of high potassium and/or pulmonary edema. We chose to gradually reduce the frequency of dialysis treatment. This seemed safe due to the close monitoring of the patient's renal function by the nurses and doctors at the dialysis department. The kidney function of our patient is now, 18 months later, in the CKD 4 stage. There is no specific treatment for his original disease and he still needs four different antihypertensive drugs, his blood pressure is depressed, and he has no significant albuminuria. It could be that he already had had a transplant, but the necessary investigations before a patient is accepted for transplantation delayed the procedure, which in this case seemed correct. This observation has been made by others [6].

A third group with recovery was autoimmune diseases and here probably immunosuppressive treatment had importance for the recovery procedure. Spontaneous remissions are found in HUS and membranous glomerulonephritis, which were also identified in the recovery group. The fact that HUS can take a long time before recovery has been described by Brunner et al., who reported two children where recovery occurred after 5 and 7 years and recommended caution before transplantation in this group [7].

Our message is that recovery of renal function could occur even after relatively long time on dialysis. It should particularly be expected in patients with relatively large or increasing urine volumes. Close monitoring and easy access to acute dialysis facilitate the decision to withdraw regular dialysis treatment. For patients who ask whether dialysis treatment is life long, the answer is that a few may recover from renal function and may stop dialysis, even after a relatively long time on dialysis treatment.

## Figures and Tables

**Table 1:** Characteristics of the patient in whom dialysis was withdrawn after 16 months of RRT.

Time	Nov. 2006	May 2007	Jan. 2008	Mar. 2008	May 2008	Sep. 2008	Feb. 2009	Mar. 2009	June 2009	Oct. 2009
s-crea *μ*mol/L		397	367		406	509	386	278	309	212

s-urea mmol/L					34.4	33.4	40.5	21.2	25	

Cystatin C-based eGFR mL/min/1.73 m^2^ body surface					24	16	17	21	20	24

Iohexol Clearance mL/min/1.73 m^2^ body surface		9.7	13		16			21		23

Hemodialysis	X Start	X From 3 to 2 times/week	X From 2 to 1 time/week	Stop		X 3 acute sessions	X 1 acute session			

**Table 2:** Characteristics of patients in the Swedish registry who recovered from renal function after more than 365 days on regular dialysis.

Diagnosis	Number	Dialysis treatment time (days)	Age (years) at start of dialysis treatment
Renovascular disease with hypertension	8	1126, 989, 821, 514, 763, 593, 475, 558	79, 73, 75, 50, 53, 75, 54, 67
Chronic renal failure NUD	7	2081, 1117, 852, 1082, 483, 478, 417	71, 79, 72, 70, 71, 44, 63
Tubular/Cortical necrosis	4	654, 641, 567, 383	45, 58, 67, 61
HUS	4	496, 801, 420, 583	82, 49, 6, 43
Crescent GN	2	1506, 408	77, 67
Cholesterol embolism	2	1102, 621	57, 72
SLE	2	995, 496	21, 29
Polyarteritis	2	710, 444	75, 71
Sclerodermia	2	484, 664	41, 52
GN membranous 1, other 1	2	1415, 464	71, 59
Wegener	1	471	56
Diabetes type ll	1	401	70
IN	1	630	86
Amyloidosis	1	624	44

## References

[1] Agraharkar M, Nair V, Patlovany M (2003). Recovery of renal function in dialysis patients. *BMC Nephrology*.

[2] Schmitt R, Coca S, Kanbay M, Tinetti ME, Cantley LG, Parikh CR (2008). Recovery of kidney function after acute kidney injury in the elderly: a systematic review and meta-analysis. *American Journal of Kidney Diseases*.

[3] Craven A-M, Hawley CM, McDonald SP, Rosman JB, Brown FG, Johnson DW (2007). Predictors of renal recovery in Australian and New Zealand end-stage renal failure patients treated with peritoneal dialysis. *Peritoneal Dialysis International*.

[4] MacDonald JA, McDonald SP, Hawley CM (2009). Recovery of renal function in end-stage renal failure—comparison between peritoneal dialysis and haemodialysis. *Nephrology Dialysis Transplantation*.

[5] Yaqoob M, McClelland P, Ahmad R (1991). Delayed recovery of renal function in patients with acute renal failure due to accelerated hypertension. *Postgraduate Medical Journal*.

[6] Siddiqui S, Norbury M, Robertson S, Almond A, Isles C (2008). Recovery of renal function after 90 d on dialysis: implications for transplantation in patients with potentially reversible causes of renal failure. *Clinical Transplantation*.

[7] Brunner K, Bianchetti MG, Neuhaus TJ (2004). Recovery of renal function after long-term dilaysis in hemolytic uremic syndrome. *Pediatr Neprhol*.

